# HIIT Ameliorates Inflammation and Lipid Metabolism by Regulating Macrophage Polarization and Mitochondrial Dynamics in the Liver of Type 2 Diabetes Mellitus Mice

**DOI:** 10.3390/metabo13010014

**Published:** 2022-12-21

**Authors:** Yin Wang, Yifan Guo, Yingying Xu, Wenhong Wang, Shuzhao Zhuang, Ru Wang, Weihua Xiao

**Affiliations:** 1 Shanghai Frontiers Science Research Base of Exercise and Metabolic Health, Shanghai University of Sport, Shanghai 200438, China; 2 The Key Lab of Exercise and Health Sciences of Ministry of Education, Shanghai University of Sport, Shanghai 200438, China; 3 Department of Rehabilitation Medicine, Shanghai Sixth People’s Hospital Affiliated to Shanghai Jiao Tong University School of Medicine, Shanghai 200233, China

**Keywords:** type 2 diabetes mellitus, high-intensity interval training, liver inflammation, liver macrophages, hepatic steatosis, mitochondria

## Abstract

High-intensity interval training (HIIT), a new type of exercise, can effectively prevent the progression of metabolic diseases. The aim of this study was to investigate the effects of HIIT on liver inflammation and metabolic disorders in type 2 diabetes mellitus (T2DM) mice induced by a high-fat diet (HFD) combined with streptozotocin (STZ) and to explore the possible mechanisms of macrophage polarization and mitochondrial dynamics. Our results showed that HIIT can increase fatty acid oxidation-related gene (PPARα, CPT1α, and ACOX1) mRNA levels and decrease adipogenesis-related gene (PPARγ) mRNA levels to improve liver metabolism in T2DM mice. The improvement of lipid metabolism disorder may occur through increasing liver mitochondrial biosynthesis-related genes (PGC-1α and TFAM) and restoring mitochondrial dynamics-related gene (MFN2 and DRP1) mRNA levels. HIIT can also reduce the mRNA levels of liver inflammatory factors (TNF-α, IL-6, and MCP-1) in T2DM mice. The reduction in liver inflammation may occur through reducing the expression of total macrophage marker (F4/80) and M1 macrophage marker (CD86) mRNA and protein and increasing the expression of M2 macrophage marker (CD163, CD206, and Arg1) mRNA and protein in the liver. HIIT can also increase the expression of insulin signaling pathway (IRS1, PI3K, and AKT) mRNA and protein in the liver of T2DM mice, which may be related to the improvements in liver inflammation and lipid metabolism. In conclusion, these results suggested that 8 weeks of HIIT can improve inflammation and lipid metabolism disorders in the liver of type 2 diabetes mellitus mice, macrophage M1/M2 polarization, and mitochondrial dynamics may be involved in this process.

## 1. Introduction

Type 2 diabetes mellitus (T2DM) is a common chronic metabolic disease worldwide. It not only results in a huge economic burden but also increases mortality. According to statistics from the International Diabetes Federation (IDF), 13.3 million people (aged 20–79 years) died of diabetic complications in 2021, and the cost of diabetes for this year was as high as 1900 billion U.S. dollars [1]. Facing the dual challenges of economy and health, T2DM is an urgent problem to be solved. Obesity is the strongest risk factor for T2DM. When the body is obese, macrophages, neutrophils, and other immune cells reside or infiltrate metabolic organs, causing chronic inflammation and metabolic disorders, thus decreasing insulin sensitivity and leading to the occurrence of insulin resistance (IR) and T2DM [2]. Therefore, targeting inflammatory pathways to prevent and treat T2DM has become a key strategy.

The liver is one of the most important metabolic organs. It has many functions, such as hepatic glycogen synthesis and decomposition, gluconeogenesis, lipid production, and oxidation. It is an important organ for maintaining the balance of glucose and lipid metabolism. In C57BL/6J mice fed a high-fat diet (HFD) for 3 days, steatosis, insulin resistance, and chronic inflammation first appeared in the liver, followed by other peripheral tissues, which proved that the chronic inflammation and metabolic changes of the liver play important roles in the pathogenesis of T2DM [3].

Liver macrophages are composed of two different macrophage populations: resident Kupffer cells (KCs) and recruited bone marrow-derived macrophages. KCs account for 80~90% of liver macrophages and are the main cells producing inflammatory mediators. KC activation produces a large number of inflammatory factors, such as tumor necrosis factor-α (TNF-α), interleukin (IL)-1β, and IL-6, and ultimately leads to chronic inflammation of the liver [4,5]. In addition, depletion of liver KCs can significantly reduce liver inflammation in obese mice, indicating that KCs play a key role in the development of chronic liver inflammation [6].

Liver lipid metabolism includes fatty acid intake, synthesis, and oxidation. When the body is overnourished, the metabolic balance of the body is broken, the liver fatty acid intake increases, oxidation decreases, and the increase in triglyceride (TG) content leads to the accumulation of fat in hepatocytes in the form of lipid droplets. Mitochondria are the organelles with the most important productivity in hepatocytes. They can also act as key controllers for the removal of fatty acids and inhibit excessive fat accumulation in hepatocytes [7]. When the body′s lipid accumulation is excessive, the structure and function of mitochondria are abnormal, resulting in reduced mitochondrial fusion, increased fission, downregulation of biosynthesis, inhibition of autophagy, and other effects [8]. Many studies have shown that mitochondrial dysfunction can reduce the activity of respiratory chain complexes and mitochondrial fatty acid β oxidation, promote the excessive production of reactive oxygen species (ROS) and lipid peroxidation, further aggravate the accumulation of fat in the liver, cause inflammation, and lead to liver insulin resistance [9,10,11,12].

As a new treatment, exercise plays an important role in the prevention and treatment of metabolic diseases. Scientific exercise can not only inhibit the progression of T2DM but also effectively prevent complications. In recent years, high-intensity interval training (HIIT) has become one of people′s favorite sports because of its high efficiency, time savings, and good compliance [13]. The American College of Sports Medicine (ACSM) refers to any form of intermittent training with an intensity of 64–90% maximum oxygen uptake as HIIT [14]. A meta-analysis showed that HIIT significantly reduced fasting blood glucose, HbA1c and IR in T2DM patients compared with the sedentary group [15]. In addition, compared with moderate intensity continuous exercise, HIIT is more effective in controlling fasting blood glucose and improving IR in T2DM patients [15]. Taking the OLETF (spontaneous type 2 diabetes animal model) rat model as the research object, we confirmed that HIIT can improve liver inflammation and IR [16]. Therefore, we established a T2DM mouse model induced by HFD combined with streptozotocin (STZ) to explore the effects of HIIT on liver inflammation and lipid metabolism in T2DM mice. We hypothesized that HIIT may improve liver inflammation and lipid metabolism by regulating hepatic macrophage polarization and mitochondrial dynamics in T2DM mice. Our findings deepen the understanding of the potential molecular mechanism by which HIIT improves T2DM liver inflammation and lipid metabolism and provides a theoretical basis for HIIT in clinical T2DM patients.

## 2. Materials and Methods

### 2.1. Animals

Male C57BL/6J mice (5 weeks) were purchased from the Model Animal Research Center of Nanjing University (Nanjing, China). The animals were housed in a temperature-controlled environment (21 ± 2 °C; 45−50% humidity) with a 12-h light–dark cycle and received water and food ad libitum. After one week of adaptive feeding, the mice were randomly divided into two groups: a normal chow diet group (NC group, n = 11) and a high-fat diet group (HFD group). The mice in the NC group were fed a standard diet (24.6% protein, 12.5% fat, 62.9% carbohydrate; D12450J, Research Diet, SYSE Ltd., Jiangsu, China). The mice in the HFD group were fed a high-fat diet (20% protein, 60% fat, 20% carbohydrate; D12492, Research Diet, SYSE Ltd., Jiangsu, China) for 12 weeks. The experimental scheme was reviewed and approved by the Animal Experiment Ethics Committee of Shanghai University of Sports (Approval no. 2016006).

### 2.2. Type 2 Diabetes Mellitus Mouse Model

The scheme comes from previous research [17,18] and was slightly modified. After 12 weeks of a high-fat diet, the mice were injected with STZ (STZ dissolved in pH = 4.5 citrate buffer) at a dose of 10 mL/kg to induce the diabetes model. The mice in the NC group were injected with the same volume of citrate buffer. One week after injection, a fasting blood glucose (FBG) test, a glucose tolerance test (GTT), and an insulin tolerance test (ITT) were performed using the tail tip blood of mice with a glucometer (Roche). The FBG in the model group was >13.8 mmol/L, which indicated successful establishment of the diabetic model [18]. A total of 23 mice reached the modeling standard, 5 failed to meet the standard, and the modeling rate was 82.4%. Subsequently, T2DM mice were randomly assigned to a T2DM-sedentary group (SED group, n = 11) and a T2DM with high-intensity interval training group (HIIT group, n = 12).

### 2.3. High-Intensity Interval Training Protocol

The HIIT group received high-intensity interval training on a running platform with a 15° incline for 8 weeks, 5 days a week (Sunday to Thursday). During exercise, the SED and HIIT groups continued to maintain a high-fat diet. Before formal exercise every day, the HIIT group was given warm-up training at a speed of 5 m/min for 10 min, followed by high-intensity training for 4 min/time and rest for 2 min/time for 10 consecutive groups/day. The speed gradually increased from 16 to 26 m/min, increased by 2 m/min every week in the first four weeks, then increased by 1 m/min every week, and finally reached 26 m/min [19].

### 2.4. Fasting Blood Glucose, Intraperitoneal Glucose Tolerance Test, and Insulin Tolerance Test

Before the fasting blood glucose (FBG) test and the intraperitoneal glucose tolerance test (IPGTT) were performed, mice were fasted overnight but could drink freely during the period. After fasting, blood from the mouse tail tip was taken to detect FBG, and then a glucose solution (dose: 1 g/kg; concentration: 0.1 g/mL) was injected intraperitoneally to detect IPGTT. For the insulin tolerance test (ITT), the mice were fasted 4 h before the test but drank freely during the period and were then injected with insulin solution (dose: 0.75 U/kg; concentration: 3.75 U/mL) intraperitoneally. The blood glucose levels in the IPGTT and ITT were measured at 0, 15, 30, 60, 90, and 120 min after injection. The areas under the curve (AUCs) of GTT and ITT were calculated by GraphPad Prism, which can more intuitively reflect the levels of glucose and insulin tolerance [20].

### 2.5. Body Composition

Twenty-four hours after the last exercise, the mice were fasted for 12 h and weighed, the mice were weighed, and the total fat mass was evaluated using Echo MRI (Echo Medical Systems, Houston, TX, USA). Percent fat mass = (fat mass/body weight) × 100%.

### 2.6. Tissue Collection and Handling Procedures

The eyeballs of mice were removed, and blood was taken. Then, the mice were dissected, and liver tissue was collected. After weighing and photographing, the liver tissue was quickly frozen in liquid nitrogen and then transferred to −80 °C for preservation. The blood samples were kept in a 4 °C refrigerator for 30 min and then centrifuged (15 min, 3000 rpm). The separated serum was transferred to a new centrifuge tube and stored at −80 °C.

### 2.7. Hematoxylin and Eosin Staining

Liver tissue (n = 3, three liver tissues from three different mice in each group) was fixed in 4% paraformaldehyde for 24 h, dehydrated, cleared, soaked in wax, and embedded in paraffin. Paraffin sections with a thickness of 4 µm were stained according to the hematoxylin and eosin (H&E) staining instructions (Servicebio, Wuhan, China) to observe the liver morphology. Under the bright-field mode of a Labophot-2 microscope (Nikon Corporation, Tokyo, Japan), the cells were observed and photographed at a magnification of 200×.

### 2.8. Oil Red O Staining

Fresh liver tissue was wrapped in optimal cutting temperature (OCT) compound (Sakura FineTek USA, Torrance, CA, USA) and frozen. Frozen sections (15 µm thick) were stained according to the Oil Red O staining instructions (Solarbio Science Technology, Beijing, China) to evaluate liver lipid deposition. Under the bright-field mode of an Olympus microscope (Tomy Corporation, Tokyo, Japan), the cells were observed and photographed at a magnification of 200×.

### 2.9. Immunofluorescence

Paraffin sections of liver tissue were dewaxed, heated in sodium citrate antigen repair solution (Beyotime Biotechnology, Haimen, China) in a 95 °C water bath for 15 min, and then cooled to room temperature. The sections were washed with PBS (*p* = 7.4, 3 times × 5 min). After blocking with blocking solution at room temperature for 2 h, the cells were incubated with primary antibodies against CD68 (1:3000, Servicebio, Wuhan, China), CD86 (1:200, Servicebio, Wuhan, China) and CD163 (1:200, Servicebio, Wuhan, China) at 4 °C overnight. The sections were washed with PBS (3 times × 5 min) and incubated with Alexa Fluor 488-conjugated goat anti-rabbit (1:500, Servicebio, Wuhan, China) and HRP-conjugated goat anti-rabbit (1:500, Servicebio, Wuhan, China) secondary antibodies at room temperature for 50 min without light. The sections were washed and incubated with DAPI (Servicebio, Wuhan, China) for 10 min. The sections were washed, and an autofluorescence quencher (Servicebio, Wuhan, China) was added for 5 min and then flushed with running water for 10 min. Finally, the sections were mounted with anti-fluorescence quenching sealing agent (Servicebio, Wuhan, China). The sections were observed with a laser scanning confocal microscope (Zeiss LSM700), and photos were taken at a magnification of 400×. Three to five visual fields were selected for each slice to take photos, analyze the positive cell number in each visual field with ImageJ software, and calculate the average value of the positive cell number of each slice.

### 2.10. Western Blot

For western blotting, total protein samples were extracted from liver tissues using RIPA lysis buffer (291 μL of RIPA, 3 μL of phenylmethyl sulfonyl fluoride, and 6 μL of protease and phosphatase inhibitor cocktail in a final volume of 300 μL), and the total protein concentrations were determined using a BCA protein assay kit (P0010S, Beyotime Institute of Biotech, Shanghai, China). A total of 40 μg extracted protein samples were separated in 10% or 7.5% SDS–PAGE gels (PG111, PG112, EpiZyme, Shanghai, China) and then transferred to PVDF membranes (Immobilon-P; Millipore, Bedford, MA, USA). After blocking with 5% nonfat dry milk (TBST make-up) at room temperature for 2 h, the PVDF membranes were incubated with related primary antibodies at 4 °C for 12–18 h and then washed and incubated with the corresponding secondary antibodies (CST, Beverly, MA, USA) for 2 h. The primary antibodies used for Western blotting were carnitine palmitoyl transferase-1 alpha (CPT1α, 97361, CST, Beverly, MA, USA, 1:1000), arginase 1 (Arg1, 93,668, CST, Beverly, MA, USA, 1:1000), protein kinase B (AKT, 4691, CST, Beverly, MA, USA, 1:1000), phosphorylated AKT (pAKT, 4060, CST, Beverly, MA, USA, 1:2000), phosphatidylinositol 3-kinase (PI3K, 4249, CST, Beverly, MA, USA, 1:1000), phosphorylated phosphatidylinositol 3-kinase (pPI3K, 4228, CST, Beverly, MA, USA, 1:1000), and α-tubulin (2125, CST, Beverly, MA, USA, 1:10,000).

A ChemiDoc MP Imaging System (Bio–Rad, Hercules, CA, USA) was used for signal detection. The protein expression content was quantified with ImageJ software and standardized to the loading control α-tubulin [21].

### 2.11. Quantitative Real-Time PCR (qPCR) Analysis

According to the manufacturer′s instructions, total RNA was extracted from liver tissues using TRIzol reagent (Invitrogen Life Technologies, Carlsbad, CA, USA), and then the concentration of the RNA samples was quantified using a Nanodrop 2000 spectrophotometer (Thermo Fisher Scientific, San Jose, CA, USA). RNA was reverse transcribed with a RevertAid^TM^ First Strand cDNA Synthesis Kit (Thermo Fisher Scientific) to synthesize cDNA for qPCR using SYBR Green PCR Master Mix (Vazyme Biotech Co., Ltd., Nanjing, China) in StepOnePlus^TM^ (Applied Biosystems, Carlsbad, CA, USA). The mRNA expression content of target genes was standardized to GAPDH expression and was calculated with the 2^−ΔΔCT^ method [19]. The primers used for qPCR are listed in Table 1.

### 2.12. Statistical Analysis

All experimental data were processed with IBM SPSS statistics 25.0 software, and the results are shown as the mean ± standard error (mean ± SEM). The difference between two groups of data was compared by Student′s *t* test, and the difference between three groups of data was compared by one-way ANOVA. A *p* < 0.05 indicates a significant difference.

## 3. Results 

### 3.1. HIIT Improves Body Weight, Fat Mass, and Blood Glucose Control in T2DM Mice

**Figure 1:**
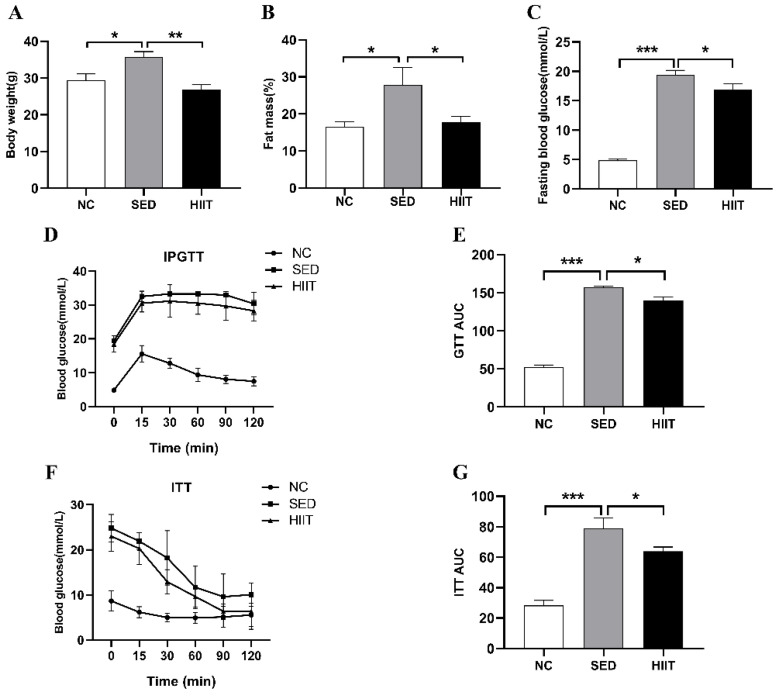
HIIT reduces hepatic body weight, fat mass, FBG, and AUCs of GTT and ITT in T2DM mice. (**A**) Body weight (n = 6 per group). (**B**) Percent fat mass (n = 6 per group). (**C**) Fasting blood glucose (n = 4 per group), (**D**) Measurement of blood glucose during glucose tolerance test (GTT) of mice (n = 5 per group), (**E**) the AUCs of GTT, (**F**) Measurement of blood glucose during insulin tolerance test (ITT) of mice (n = 5 per group), (**G**) the AUCs of ITT. Values are mean ± SEM. * *p* < 0.05, ** *p* < 0.01, *** *p* < 0.001.

Compared with the NC group, the body weight (*p* < 0.05) (Figure 1A), fat mass (*p* < 0.05) (Figure 1B), FBG, and the AUCs of GTT and ITT (*p* < 0.001) (Figure 1C–G) were significantly increased in the SED group, indicating that the T2DM mouse model was successfully established. After 8 weeks of HIIT intervention, compared with the SED group, the body weight (*p* < 0.01) (Figure 1A), fat mass (*p* < 0.05) (Figure 1B), FBG, and AUCs of GTT and ITT (*p* < 0.05) (Figure 1C–G) were significantly decreased in the HIIT group.

### 3.2. HIIT Improves Hepatic Steatosis and Fat Content in T2DM Mice

**Figure 2:**
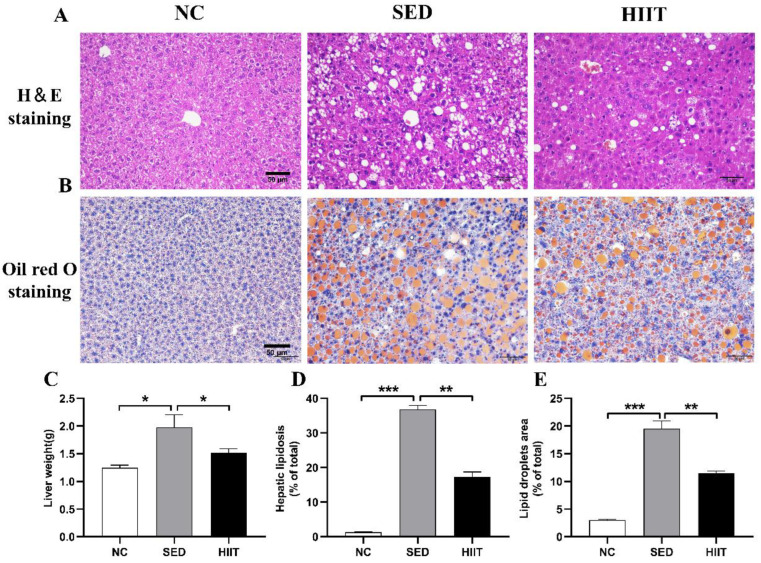
HIIT reduces hepatic fat content in T2DM mice. (**A**) Hematoxylin and eosin (H&E) staining of the liver. (**B**) Oil Red O staining of the liver. (**C**) Liver weight (n = 4 per group). (**D**) Hepatic lipid deposition (n = 4 per group). (**E**) Liver lipid droplet area (n = 4 per group). The black scale is 50 μm. Values are mean ± SEM. * *p* < 0.05, ** *p* < 0.01, *** *p* < 0.001.

Compared with the NC group, the liver weight of the SED group (NC: 1.24 ± 0.03 g vs. SED: 1.97 ± 0.12 g) increased significantly, while HIIT decreased the liver weight of T2DM mice (HIIT: 1.59 ± 0.06 g) (Figure 2C). To detect the effect of HIIT on hepatic steatosis and lipid deposition in T2DM mice, we stained the mouse livers with H&E and Oil Red O and statistically analyzed the hepatic fat vacuole area and lipid droplet area. The results showed that compared with the NC group, the steatosis and lipid droplet area of the liver in the SED group increased significantly (*p* < 0.001); compared with the SED group, the numbers and sizes of hepatic fat vacuoles and lipid droplets in the HIIT group were significantly decreased (Figure 2A,B,D,E).

### 3.3. HIIT Improves Liver Insulin Resistance in T2DM Mice

**Figure 3:**
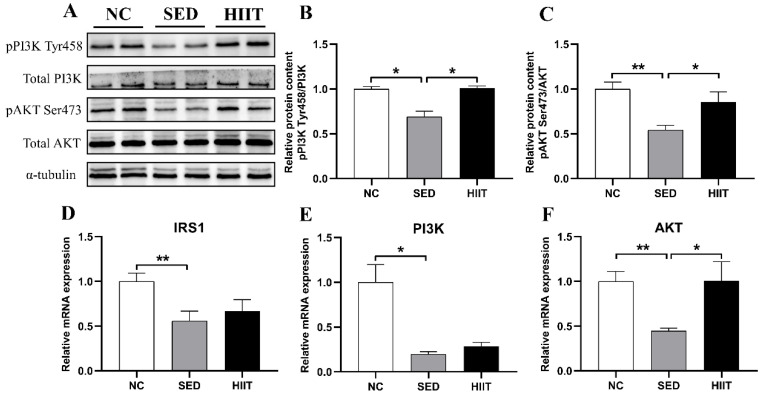
HIIT improves liver insulin resistance in T2DM mice. (**A**) Protein expression of pPI3K and pAKT and the internal control α-tubulin in the liver. (**B**,**C**) Quantification of the proteins described in (**A**) with normalization to the protein levels of α-tubulin (n = 4 per group). (**D**–**F**) The mRNA expression levels of IRS1, PI3K, and AKT (n = 8 per group). Values are mean ± SEM. * *p* < 0.05, ** *p* < 0.01.

The liver insulin IRS1/PI3K/AKT signaling pathway is of great significance in improving hepatic insulin resistance. To verify the improvement effect of HIIT on insulin resistance in T2DM mice, we detected the mRNA and protein expression levels of IRS1/PI3K/AKT, and the results were consistent with our expectations. Compared with the NC group, the mRNA levels of IRS1 (0.56-fold, *p* < 0.01), PI3K (0.20-fold, *p* < 0.05) and AKT (0.45-fold, *p* < 0.01) (Figure 3D–F) in the SED group were significantly decreased, and the protein levels of phosphorylation-PI3K (Tyr458) (0.69-fold, *p* < 0.05) (Figure 3A,B) and phosphorylation-AKT (Ser473) (0.54-fold, *p* < 0.01) (Figure 3A,C) were also decreased. After 8 weeks of HIIT, the mRNA levels of IRS1 (0.67-fold), PI3K (0.28-fold), and AKT (1.0-fold, *p* < 0.05) (Figure 3D–F) were increased, and the protein expression levels of pPI3K (1.0-fold, *p* < 0.05) (Figure 3A,B) and pAKT (0.85-fold, *p* < 0.05) (Figure 3A,C) were significantly increased.

### 3.4. HIIT Improves Liver Inflammation in T2DM Mice

**Figure 4:**
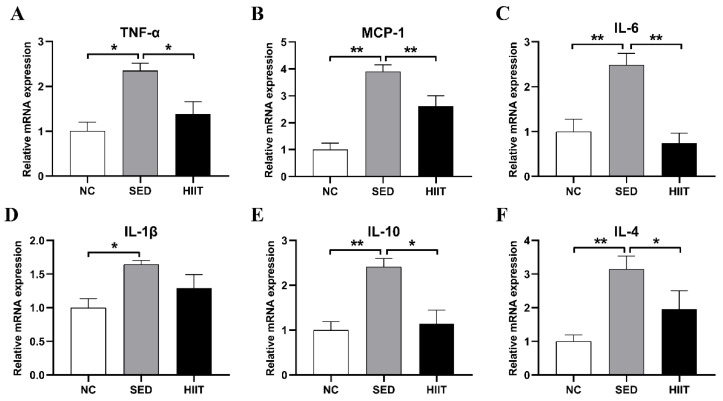
HIIT reduces liver inflammation in T2DM mice. (**A**–**F**) The mRNA expression levels of TNF-α, MCP-1, IL-6, IL-1β, IL-10, and IL-4. Values are mean ± SEM (n = 8 per group). * *p* < 0.05, ** *p* < 0.01.

The liver inflammatory infiltration of T2DM mice induced by HFD increased significantly. However, HIIT for 8 weeks significantly decreased inflammatory infiltration. As shown, compared with the NC group, the mRNA levels of the pro-inflammatory cytokines TNF-α (2.3-fold, *p* < 0.05), monocyte chemoattractant protein-1 (MCP-1) (3.9-fold, *p* < 0.01), IL-6 (2.48-fold, *p* < 0.01), and IL-1β (1.64-fold, *p* < 0.05) (Figure 4A–D), and the anti-inflammatory cytokines IL-10 (2.4-fold, *p* < 0.01) and IL-4 (3.15-fold, *p* < 0.01) (Figure 4E,F) in the SED group were significantly increased. After exercise, the mRNA levels of the pro-inflammatory cytokines TNF-α (1.38-fold, *p* < 0.05), MCP-1 (2.6-fold, *p* < 0.01), and IL-6 (0.74-fold, *p* < 0.01) (Figure 4A–C) and the anti-inflammatory cytokines IL-10 (1.14-fold, *p* < 0.05) and IL-4 (1.96-fold, *p* < 0.05) (Figure 4E,F) in the HIIT group were significantly decreased.

### 3.5. HIIT Decreases the Pro-Inflammatory Polarization of Liver Macrophages in T2DM Mice

**Figure 5:**
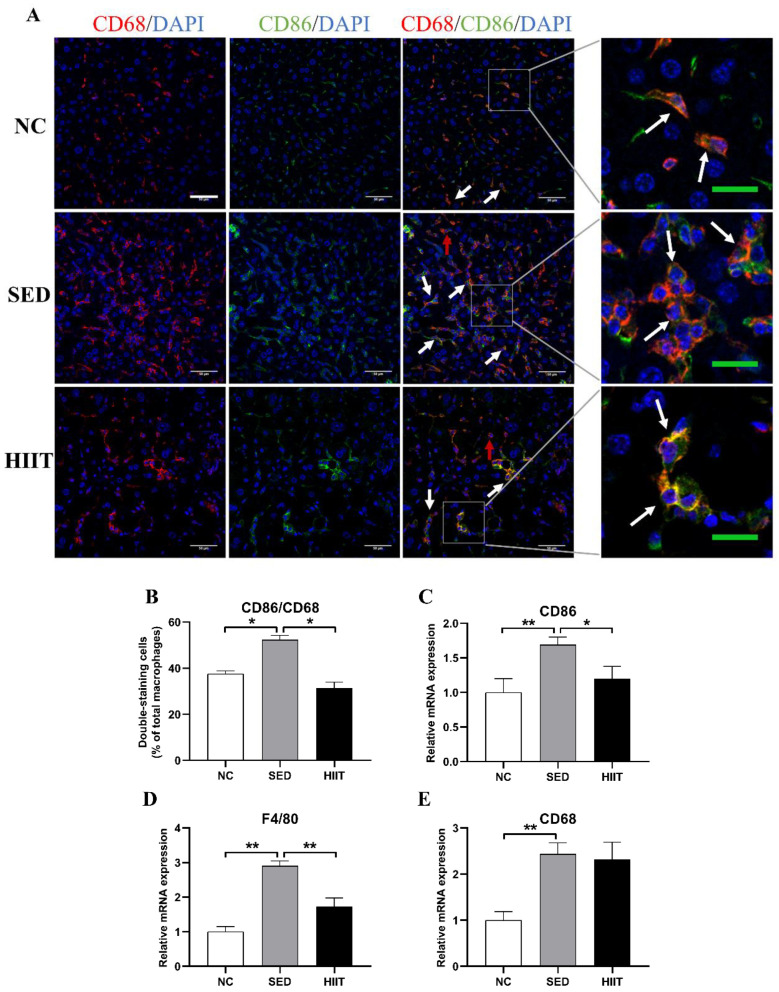
HIIT decreases the pro-inflammatory polarization of liver macrophages in T2DM mice. (**A**) CD86/CD68 (M1) immunofluorescence double labeling results. (**B**) Percentage of CD86/CD68-positive cells (n = 3 per group). (**C**–**E**) The mRNA expression of CD86, F4/80 and CD68 (n = 8 per group). The white scale is 50 μm. The green scale is 20 μm. The red arrow indicates CD68 single-positive cells. The white arrow indicates CD86 and CD68 double-positive cells. Values are mean ± SEM. * *p* < 0.05, ** *p* < 0.01.

In metabolic diseases, liver macrophages are particularly important for the occurrence and maintenance of liver inflammation. Therefore, this study evaluated liver macrophage markers. Immunofluorescence results showed that, compared with the NC group, the number of pro-inflammatory macrophages (M1) cells in the SED group increased significantly, and HIIT significantly decreased the number of M1 macrophages (Figure 5A,B). At the transcriptional level, compared with the NC group, the mRNA expression levels of the total macrophage markers F4/80 (2.9-fold, *p* < 0.01) and CD68 (2.43-fold, *p* < 0.01) increased significantly (Figure 5D,E), and the mRNA expression of the M1 macrophage marker CD86 (1.69-fold, *p* < 0.01) increased significantly in the SED group (Figure 5C). After 8 weeks of HIIT, the mRNA expression levels of CD86 (1.2-fold, *p* < 0.05) and F4/80 (1.7-fold, *p* < 0.01) decreased significantly (Figure 5C,D).

### 3.6. HIIT Increases the Anti-Inflammatory Polarization of Liver Macrophages in T2DM Mice

**Figure 6:**
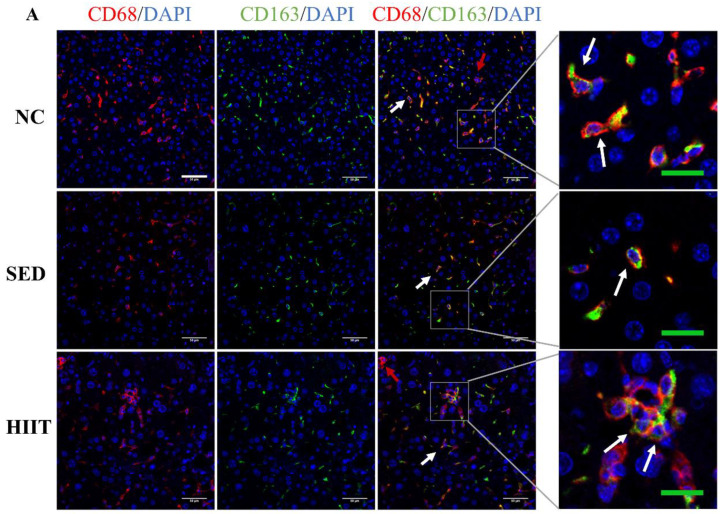

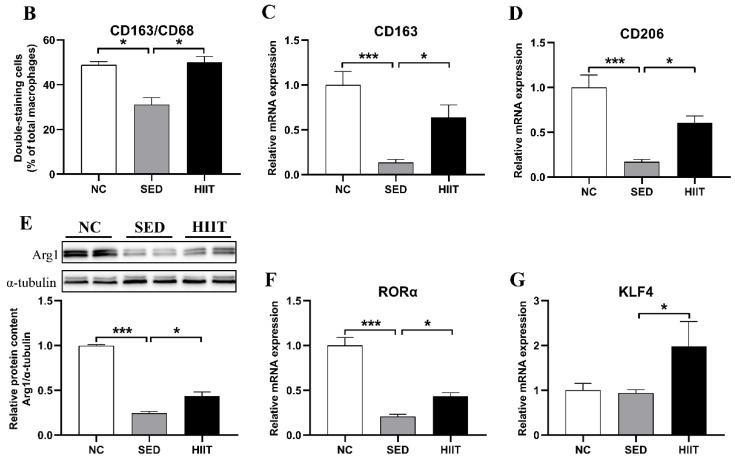
HIIT increases the anti-inflammatory polarization of liver macrophages in T2DM mice. (**A**) CD163/CD68 (M2) immunofluorescence double-labeling results. (**B**) Percentage of CD163/CD68-positive cells (n = 3 per group). (**E**) Protein expression of Arg1 and the internal control α-tubulin in the liver (n = 4 per group). (**C**,**D**,**F**,**G**) The mRNA expression levels of CD163, CD206, RORα and KLF4 (n = 8 per group). The white scale is 50 μm. The green scale is 20 μm. The red arrow indicates CD68 single-positive cells. The white arrow indicates CD163 and CD68 double-positive cells. Values are mean ± SEM. * *p* < 0.05, *** *p* < 0.001.

The immunofluorescence results of this study showed that (Figure 6A,B), compared with the NC group, the number of anti-inflammatory macrophages (M2) in the SED group decreased significantly, while HIIT significantly increased the number of M2 macrophages. From the results of Western blot and qPCR, the expression of Arg1 protein (Figure 6E) and the expression levels of the M2 macrophage markers CD163 (0.13-fold, *p* < 0.01) and CD206 (0.17-fold, *p* < 0.01) mRNA (Figure 6C,D) in the SED group were significantly lower than those in the NC group. After 8 weeks of HIIT, the protein expression of Arg1 (Figure 6E) and the mRNA expression levels of CD163 (0.69-fold, *p* < 0.05) and CD206 (0.60-fold, *p* < 0.01) (Figure 6C, D) increased significantly.

Retinoid acid receptor related orphan receptor α (RORα) is a key regulator of M1/M2 polarization of liver macrophages. RORα can promote the M2 polarization of KCs by inducing Kruppel like factor 4 (KLF4) [22]. Compared with the NC group, the RORα (0.21-fold, *p* < 0.01) mRNA levels decreased significantly in the SED group (Figure 6F). After exercise, the gene expression levels of RORα (0.43-fold, *p* < 0.01) and KLF4 (1.98-fold, *p* < 0.01) in the HIIT group increased significantly (Figure 6F,G).

### 3.7. HIIT Improves Liver Lipid Metabolism in T2DM Mice

**Figure 7:**
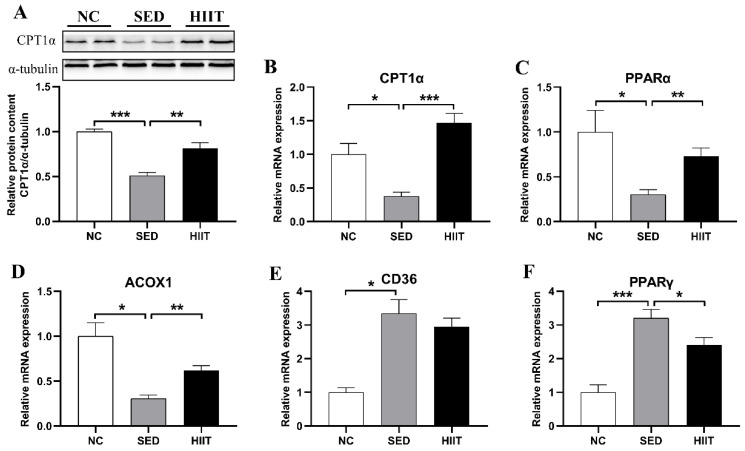
HIIT improves liver lipid metabolism in T2DM mice. (**A**) Protein expression of CPT1α and the internal control α-tubulin in the liver (n = 4 per group). (**B**–**F**) The mRNA expression levels of CPT1α, PPARα, ACOX1, CD36, and PPARγ (n = 8 per group). Values are mean ± SEM. * *p* < 0.05, ** *p* < 0.01, *** *p* < 0.001.

To evaluate the effect of 8 weeks of HIIT on liver lipid metabolism, we detected the mRNA and protein expression levels of key factors of fatty acid decomposition and synthesis. The experimental results are shown in Figure 7. Compared with the NC group, the mRNA expression levels of the key fatty acid oxidation factors CPT1α (0.38-fold, *p* < 0.05), peroxisome proliferators-activated receptor (PPAR)α (0.3-fold, *p* < 0.05), and acyl-CoA oxidase 1 (ACOX1) (0.3-fold, *p* < 0.05) (Figure 7B–D) decreased significantly, and the protein expression level of CPT1α (0.51-fold, *p* < 0.01) (Figure 7A) also decreased markedly in the SED group. Eight weeks of HIIT markedly increased the mRNA expression levels of CPT1α (1.47-fold, *p* < 0.001), PPARα (0.73-fold, *p* < 0.05), and ACOX1 (0.62-fold, *p* < 0.05) (Figure 7B–D), and the protein expression of CPT1α (0.81-fold, *p* < 0.01) (Figure 7A). Fatty acid transporters (CD36) are an important fatty acid transporter in fatty acid metabolism that mediate the uptake of fatty acids in the liver. In T2DM mice, the mRNA expression of CD36 (3.34-fold, *p* < 0.01) (Figure 7E) increased significantly, and the subsequent 8-week HIIT decreased the level of CD36 mRNA (2.95-fold, *p* > 0.05) (Figure 7E), but the decrease was not significant. PPARγ is a key regulator of adipogenesis. The mRNA expression of PPARγ (3.21-fold, *p* < 0.01) (Figure 7F) increased significantly in T2DM mice, while exercise decreased PPARγ (2.41-fold, *p* < 0.05) (Figure 7F) mRNA expression.

### 3.8. HIIT Improves Liver Mitochondrial Biosynthesis and Dynamics in T2DM Mice

**Figure 8:**
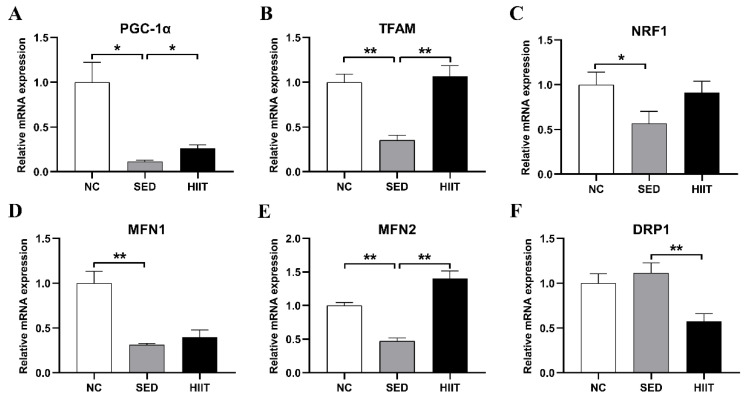
HIIT improves liver mitochondrial biosynthesis and dynamics in T2DM mice. (**A**–**F**) The mRNA expression levels of PGC-1α, TFAM, NRF1, MFN1, MFN2, and DRP1. Values are mean ± SEM (n = 8 per group). * *p* < 0.05, ** *p* < 0.01.

Mitochondria are the main organelles for fatty acid β oxidation and utilization. To study the effects of 8 weeks of HIIT on the density and number of mitochondria, we detected the mRNA levels of factors related to mitochondrial biosynthesis and dynamic changes. Compared with the NC group, the mRNA levels of the mitochondrial biosynthesis-related factors peroxisome proliferator-activated receptor γ coactivator-1α (PGC-1α) (0.11-fold, *p* < 0.05), mitochondrial transcription factor A (TFAM) (0.35-fold, *p* < 0.05), and nuclear respiratory factor 1 (NRF1) (0.57-fold, *p* < 0.05) (Figure 8A–C) were significantly lower in T2DM mice, while 8-week HIIT decreased the mRNA levels of PGC-1α (0.26-fold, *p* < 0.05), TFAM (1.06-fold, *p* < 0.05), and NRF1 (0.91-fold, *p* > 0.05) (Figure 8A–C). In T2DM mice, the mRNA levels of the mitochondrial fusion-related factors (MFN)1 (0.31-fold, *p* < 0.01) and MFN2 (0.47-fold, *p* < 0.01) (Figure 8D,E) were decreased. After 8 weeks of HIIT, the mRNA expression levels of MFN1 (0.39-fold, *p* > 0.05) and MFN2 (1.4-fold, *p* < 0.01) (Figure 8D,E) increased, and the mRNA expression of the mitochondrial fission-related factor DRP1 (0.57-fold, *p* < 0.01) (Figure 8F) decreased.

## 4. Discussion

### 4.1. HIIT Improves Insulin Resistance in T2DM Mice

Exercise has become the cornerstone of the treatment of T2DM because of its safe and effective characteristics. Many studies have proven that aerobic training alone, resistance training alone, or combined training can improve insulin sensitivity and HbA1c in T2DM patients [23,24,25,26,27]. The study found that HIIT, a new method of exercise, can achieve health effects similar to or even better than medium-intensity exercise while saving exercise time and bringing pleasure to patients [28]. In a large number of clinical studies summarized by Jimenez Maldonado et al., HIIT has extensive benefits in glucose metabolism and insulin sensitivity in patients with metabolic syndrome [29]. However, the specific mechanism by which HIIT improves T2DM is not completely clear.

After 8 weeks of HIIT, we measured the fasting blood glucose of T2DM mice and conducted glucose tolerance and insulin tolerance tests. It was found that the fasting blood glucose level and the areas under the curve of glucose and insulin tolerance of the HIIT group were significantly lower than those of the SED group [21], findings that were consistent with the study of Wang et al. They found that 8 weeks of HIIT can also improve systemic glucose homeostasis and insulin sensitivity in diet-induced obese mice [19]. The above results showed that HIIT for 8 weeks can improve glucose metabolism and insulin sensitivity in T2DM mice.

Insulin resistance is a key factor in the pathogenesis of T2DM. IRS/PI3K/AKT is the main signaling pathway mediating the insulin effect [30,31]. As one of the most sensitive target tissues of insulin, the liver plays an important role in regulating insulin-mediated glucose and lipid metabolism [32,33]. In this study, the results showed that the mRNA and protein expression levels of IRS1, PI3K, and AKT in the livers of mice in the SED group were significantly lower than those in the NC group, which was consistent with previous results [34], indicating that the hepatic insulin signaling pathway was damaged and insulin sensitivity was reduced. After 8 weeks of HIIT, the mRNA and protein expression levels of IRS1, PI3K, and AKT in the livers of HIIT group mice had increased significantly. Combined with the results of GTT and ITT, HIIT can partially restore the transmission of the hepatic insulin signaling pathway and improve insulin sensitivity and systemic glucose tolerance. The research results of da Luz et al. also showed that 8 weeks of endurance exercise can significantly increase the phosphorylation of insulin receptor, IRS and AKT in the liver of obese rats induced by HFD [35]. The study also found that 8 weeks of aerobic exercise had a similar improvement effect [36]. Therefore, 8 weeks of HIIT can improve hepatic IR in T2DM mice by upregulating the transmission of the hepatic insulin signaling pathway.

### 4.2. HIIT Improves Liver Inflammation and Regulates Macrophage M1/M2 Polarization of the Liver in T2DM Mice

Chronic low-grade inflammation is also an important pathogenic factor in T2DM patients. The liver is not only an important metabolic organ but also the tissue where inflammation occurs first in obesity. Many studies have shown that pro-inflammatory cytokines and chemokines such as TNF-α, IL-6, MCP-1, and IL-1β in the liver play key roles in the progression of metabolic diseases [37,38,39,40]. These pro-inflammatory cytokines can damage the insulin signaling pathway in target tissues, resulting in IR. Therefore, the liver inflammatory response is closely related to IR [41]. This study supports the upregulation of the gene expression of the inflammatory factors TNF-α, IL-6, MCP-1, and IL-1β in the liver of T2DM mice induced by HFD/STZ. Subsequently, 8 weeks of HIIT significantly downregulated the gene expression of inflammatory factors in T2DM mice. These results are consistent with the study of Diniz et al., in which it was reported that 8 weeks of aerobic exercise reduced the concentrations of inflammatory cytokines in the liver of NAFLD mice [36]. Pereira et al. found that short-term combined aerobic and strength training can also reduce liver TNF-α protein expression in mice with hepatic steatosis [42]. The above results show that different exercise patterns can reduce liver inflammation in diet-induced hepatic steatosis mice.

Contrary to our expectations, this study also found that the mRNA levels of the anti-inflammatory cytokines IL-10 and IL-4 in T2DM mice increased significantly. After 8 weeks of HIIT intervention, the gene expression levels of anti-inflammatory cytokines decreased significantly. Previous studies have found that there were differences in the expression level of the anti-inflammatory factor IL-10 in different tissues of the T2DM model, and the gene expression of IL-10 in liver tissue was significantly increased, which was consistent with the results of Oyenhi et al. [43,44]. The above research provides sufficient evidence for the results of this study. In addition, most reports on the effect of exercise on the liver anti-inflammatory factor IL-10 under obesity are increased, but in the study of Diniz et al., aerobic exercise was found to reduce the level of IL-10 protein in the liver of HFD-induced obese mice [39]. These reports are consistent with our results. The above studies found that the expression levels of anti-inflammatory cytokines increased or decreased with the increase or decrease in pro-inflammatory cytokines, which may be because IL-10 and IL-4, as anti-inflammatory cytokines, can regulate the activity of pro-inflammatory cytokines and produce a direct response to pro-inflammatory cytokines. Therefore, 8 weeks of HIIT can improve the hepatic inflammatory response in T2DM mice.

It is well known that obesity can lead to an imbalance in the M1/M2 polarization of liver macrophages and then cause liver inflammation. Given the relieved inflammation in the liver of T2DM-HIIT mice, we further investigated whether HIIT had an effect on the M1/M2 polarization of liver macrophages. In this study, qPCR results showed that the gene expression levels of total macrophage markers (F4/80, CD68) and an M1 marker (CD86) in T2DM mice were significantly higher than those in the NC group, and the expression levels of M2 markers (CD163, CD206) were significantly decreased. Western blotting and immunofluorescence results were consistent with the qPCR results. Previous studies also found that total macrophages and M1 macrophages increased, while M2 macrophages decreased in the liver of NAFLD mice induced by HFD [45,46]. Combined with the increase in inflammatory cytokines in this study, the results showed that liver macrophages were activated and increased the M1 polarization of macrophages in T2DM mice, thus upregulating the expression of inflammatory cytokines. Previous reports have confirmed that exercise can balance the M1/M2 polarization of liver macrophages in obese and NAFLD mice, but this phenomenon has not been reported in T2DM. In this study, T2DM mice were subjected to HIIT for 8 weeks. It was found that the gene levels of total and M1 macrophage markers decreased significantly, while M2 markers increased significantly after exercise. From the Western blotting and immunofluorescence results, HIIT increased the expression of the M2 marker (Arg1); the number of CD86/CD68-positive cells decreased significantly, and the number of CD163/CD68-positive cells increased significantly after exercise. In addition, Ai et al. found that 8 weeks of downhill running could inhibit M1 polarization and increase M2 polarization of liver macrophages in NAFLD mice [45]. Linden’s team also found the same effect after 12 weeks of HIIT with OLETF mice as a model [16]. The above results show that HIIT could improve inflammation by promoting the M2 polarization of liver macrophages in T2DM mice.

Then, we explored the mechanism by which HIIT regulates the polarization of liver macrophages and found that HIIT could significantly increase the mRNA expression levels of RORα and KLF4 in T2DM mice, while RORα-induced upregulation of KLF4 expression is one of the key mechanisms mediating M2 polarization of KCs [22]. The results suggest that HIIT may promote the M2 polarization of KCs through the RORα-dependent KLF4 pathway in T2DM mice. Because this study is based on research reports in the literature, the gene detection of the above two factors only provides a possibility. In the future, a RORα gene knockout mouse model can be established to further explore whether HIIT regulates the polarization of liver macrophages through the RORα/KLF4 signaling pathway to improve the chronic inflammation of T2DM mice.

### 4.3. HIIT Improves Liver Lipid Metabolism Disorder and Liver Mitochondrial Dynamics in T2DM Mice

Hepatic lipid deposition is a pathological phenomenon of metabolic disorder. On the one hand, hepatic lipid deposition can lead to hepatic lipotoxicity and further activate the inflammatory signaling pathway, causing hepatic inflammation; on the other hand, liver fat deposition can inhibit the insulin signaling pathway, resulting in IR [47,48,49]. In this study, a large number of fat vacuoles and large areas of red lipid droplets appeared in the liver tissue of T2DM mice. After 8 weeks of HIIT exercise, the size and number of lipid droplets in the liver tissue of T2DM mice decreased significantly. The results showed that exercise could improve the liver lipid deposition of T2DM mice. Previous studies also supported the results of this experiment. HIIT significantly reduces the liver fat content of OLETF rats, NAFLD, and obese mice induced by a HFD [16,19,45]. Therefore, HIIT can improve liver fat deposition in T2DM mice. Hepatic fat deposition is regulated by a variety of biochemical pathways, mainly involving fatty acid synthesis and catabolism. This study examined the related factors of different metabolic mechanisms; for example, PPARγ is the key factor in adipogenesis, CD36 is the key factor in fatty acid uptake, and CPT1α, PPARα, and ACOX1 are key factors in fatty acid oxidation. In this study, 8 weeks of HIIT significantly inhibited PPARγ gene expression and promoted PPARα, CPT1α, and ACOX1 gene expression in the livers of T2DM mice. Interestingly, there was no change in CD36 gene expression after exercise. This result is consistent with Linden′s study [16], in which moderate-intensity exercise and HIIT were used as interventions in diabetic rats. It was found that only moderate-intensity exercise could reduce the expression of CD36 protein, suggesting that HIIT may not reduce liver fat content by reducing CD36 expression. In addition, previous studies have found that exercise can increase PPARα and CPT1α gene levels in obese mice [42,50]. In conclusion, HIIT may improve liver fat metabolism by inhibiting liver fat production and promoting liver fatty acid oxidation to reduce liver fat content in T2DM mice.

Mitochondria are the main organelles that produce ATP in cells. Their dysfunction causes bioenergy failure, oxidative stress, inflammation, and cell death, and plays a central role in metabolic diseases [51]. The decrease in mitochondrial biosynthesis and the disruption of dynamics will lead to mitochondrial dysfunction. PGC-1α is a cotranscriptional regulator regulating mitochondrial biosynthesis. It can act together with NRF1, NRF2, TFAM, and other transcription factors to participate in the regulation of mitochondrial biosynthesis [52]. In this study, the mRNA levels of PGC-1α, NRF1, and TFAM in the livers of T2DM mice decreased significantly. Then, we treated T2DM mice with 8 weeks of HIIT and found that PGC-1α and TFAM gene expression levels increased significantly. This finding supports previous research results. For example, Heiat et al. showed that 8 weeks of continuous swimming training increased the level of PGC-1α in the livers of NAFLD mice [53]. Santos Alves et al. also found that compared with a sedentary group, 12 weeks of treadmill training significantly increased the protein levels of PGC-1α and TFAM in the livers of Sprague Dawley rats [54]. It is well known that liver mitochondrial dysfunction can lead to a decline in fatty acid oxidation and disorders of lipid metabolism. Therefore, the results of this study suggest that 8 weeks of HIIT may regulate lipid metabolism by increasing liver mitochondrial biosynthesis in T2DM mice.

In addition, the imbalance of mitochondrial dynamics can lead to its dysfunction, resulting in metabolic diseases. Mitochondrial dynamics mainly include two processes: mitochondrial fusion and fission. MFN1 and MFN2 are proteins that mediate mitochondrial outer membrane fusion. DRP1 can mediate mitochondrial fission by transferring to the mitochondrial outer membrane. The study found that the expression level of MFN2 protein decreased significantly in NASH patients, liver steatosis mice and NASH mice; another study found that MFN1 protein expression was significantly reduced in the liver of NAFLD mice [55,56]. Our study also found that the mRNA expression levels of liver MFN1 and MFN2 were significantly reduced in T2DM mice. After 8 weeks of HIIT intervention, MFN2 gene expression in the liver of T2DM mice increased significantly. Previous studies have also found that endurance exercise can upregulate the expression levels of the MFN1 and MFN2 proteins in the livers of HFD-induced NASH rats [57]. Our study also found that HIIT could significantly reduce the expression of the DRP1 gene in the livers of T2DM mice. Previous studies have found that exercise can reduce DRP1 protein phosphorylation in the skeletal muscle of obese rats [58]. The above results show that exercise can restore the dynamic balance of mitochondria by increasing mitochondrial fusion and decreasing fission in T2DM mice. The increase in mitochondrial fusion and the decrease in fission can improve the disorder of liver lipid metabolism in obese mice [59,60]. In conclusion, our study shows that 8 weeks of HIIT may improve liver lipid metabolism disorders by balancing the dynamics of liver mitochondria in T2DM mice.

## 5. Conclusions

**Figure 9:**
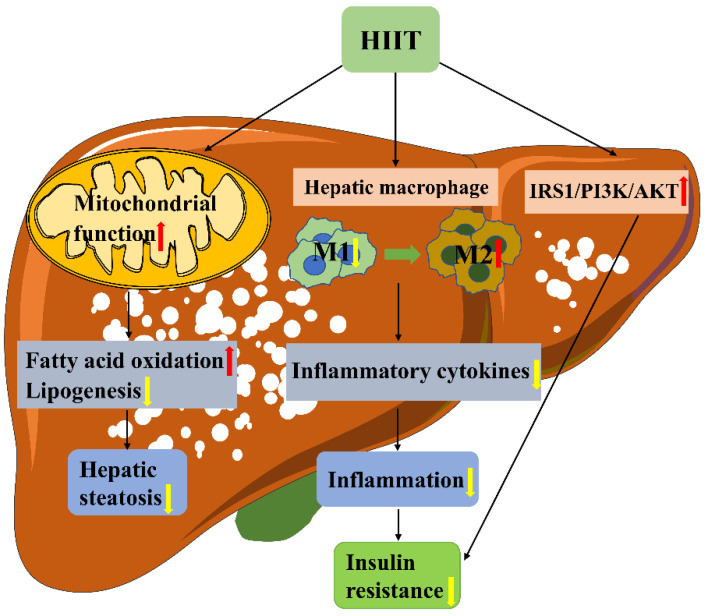
Mechanism of HIIT improving liver inflammation and insulin resistance in T2DM mice.

In conclusion, the results of this study suggest that 8 weeks of high-intensity interval training can improve inflammation and lipid metabolism disorders in the liver of type 2 diabetes mellitus mice, macrophage M1/M2 polarization, and mitochondrial dynamics may be involved in this process. However, this study is a phenomenal observation study, and the determination of causality needs further exploration. Later, we can establish a gene knockout mouse model, which will be more convincing for determining causality. However, this study still provides a theoretical basis for the formulation of an exercise plan for patients with type 2 diabetes (Figure 9).

## Figures and Tables

**Table 1:** Primer sequence.

Gene Name	Forward Primer Sequences	Reverse Primer Sequences
MCP-1	5′-GCTCAGCCAGATGCAGTTAAC-3′	5′-CTCTCTCTTGAGCTTGGTGAC-3′
PGC-1α	5′-GAGTGTGCTGCTCTGGTTGG-3′	5′-AATATGTTCGCAGGCTCATTGTTG-3′
ACOX1	5′-CCAATGCTGGTATCGAAGAATG-3′	5′-CGACTGAACCTGGTCATAGATT-3′
PPARα	5′-AGGGCCTCCCTCCTACGCTTG-3′	5′-GGGTGGCAGGAAGGGAACAGA-3′
AKT	5′-GCCGCTACTATGCCATGAAGATCC-3′	5′-GCAGGACACGGTTCTCAGTAAGC-3′
IRS1	5′-CCAGCAGCAGTAGCAGCATCAG-3′	5′-GCTTACCGCCACCACTCTCAAC-3′
PI3K	5′-CCTTGGAGTGGTTTGACCATTA-3′	5′-CATCCCAGCTTAATGCTGTATCTATC-3′
MFN1	5′-CCATCTTTCAGGTCCCTAGATC-3′	5′-GCTCCGTACATACTTAAGGTGA-3′
MFN2	5′-GCATTCTTGTGGTCGGAGGAGTG-3′	5′-TGGTCCAGGTCAGTCGCTCATAG-3′
DRP1	5′-ACTGATTCAATCCGTGATGAGT-3′	5′-GTAACCTATTCAGGGTCCTAGC-3′
IL-10	5′-CAAGGAGCATTTGAATTCCC-3′	5′-GGCCTTGTAGACACCTTGGTC-3′
CD68	5′-CAAAGCTTCTGCTGTGGAAAT-3′	5′-GACTGGTCACGGTTGCAAG-3′
CPT1α	5′-AGCCAGACTCCTCAGCAGCAG-3′	5′-CACCATAGCCGTCATCAGCAAC-3′
IL-6	5′-CAGCCACTGCCTTCCCTACT-3′	5′-CAGTGCATCAT CGCTGTTCAT-3′
IL-1β	5′-GAAATGCCACCTTTTGACAGTG-3′	5′-TGGATGCTCTCATCAGGACAG-3′
CD206	5′-CTCTGTTCAGCTATTGGACGC-3′	5′-CGGAATTTCTGGGATTCAGCTTC-3′
CD86	5′-GCCGTGCCCATTTACAAAGG-3′	5′-GTTCCTGTCAAAGCTCGTGC-3′
CD163	5′-GCAAAAACTGGCAGTGGG-3′	5′-GTCAAAATCACAGACGGAGC-3′
F4/80	5′-GAATCTTGGCCAAGAAGAGAC-3′	5′-GAATTCTCCTTGTATATCATCAGC-3′
PPARγ	5′-CCAAGAATACCAAAGTGCGATC-3′	5′-TCACAAGCATGAACTCCATAGT-3′
RORα	5′-CTTCTTCCCCTACTGTTCCTTC-3′	5′-TCTCTGCTTGTTCTGGTAGTTT-3′
KLF4	5′-ACCTCCTGGACCTAGACTTTAT-3′	5′-GAAGACGAGGATGAAGCTGAC-3′
TFAM	5′-GGAATGTGGAGCGTGCTAAAA-3′	5′-TGCTGGAAAAACACTTCGGAATA-3′
NRF1	5′-GTTGCCCAAGTGAATTACTCTG-3′	5′-TCGTCTGGATGGTCATTTCAC-3′
IL-4	5′-TACCAGGAGCCATATCCACGGATG-3′	5′-TGTGGTGTTCTTCGTTGCTGTGAG-3′
CD36	5′-GCAGGTCTATCTACGCTGTGTTCG-3′	5′-TGTCTGGATTCTGGAGGGGTGATG-3′
TNF-α	5′-CTTCTGTCTACTGAACTTCGGG-3′	5′-CACTTGGTGGTTTGCTACGAC-3′
GAPDH	5′-ACTCCACTCACGGCAAATTC-3′	5′-TCTCCATGGTGGTGAAGACA-3′

## Data Availability

The data that support the findings of this study are available from the corresponding author upon reasonable request due to privacy or ethical restrictions.
